# Innovative imaging of insulinoma: the end of sampling? A review

**DOI:** 10.1530/ERC-19-0476

**Published:** 2020-01-17

**Authors:** Emanuel Christ, Kwadwo Antwi, Melpomeni Fani, Damian Wild

**Affiliations:** 1 Division of Endocrinology, Diabetology and Metabolism, University Hospital of Basel, University of Basel, Basel, Switzerland; 2 Center for Neuroendocrine and Endocrine Tumors, University Hospital Basel, Basel Switzerland; 3 Clinic of Radiology and Nuclear Medicine, University Hospital, Basel, Switzerland

**Keywords:** glucagon-like peptide-1 receptor, endogenous hyperinsulinemic hypoglycemia, insulinoma, multiple neuroendocrine neoplasia type-1, ^111^In-DOTA/DTPA-exendin-4 SPECT/CT, ^99m^Tc-HYNIC-exendin-4 SPECT/CT, ^68^Ga-DOTA-exendin-4 PET/CT

## Abstract

Receptors for the incretin glucagon-like peptide-1 (GLP-1R) have been found overexpressed in selected types of human tumors and may, therefore, play an increasingly important role in endocrine gastrointestinal tumor management. In particular, virtually all benign insulinomas express GLP-1R in high density. Targeting GLP-1R with indium-111, technetium-99m or gallium-68-labeled exendin-4 offers a new approach that permits the successful localization of small benign insulinomas. It is likely that this new non-invasive technique has the potential to replace the invasive localization of insulinomas by selective arterial stimulation and venous sampling. In contrast to benign insulinomas, malignant insulin-secreting neuroendocrine tumors express GLP-1R in only one-third of the cases, while they more often express the somatostatin subtype 2 receptors. Importantly, one of the two receptors appears to be always overexpressed. In special cases of endogenous hyperinsulinemic hypoglycemia (EHH), that is, in the context of MEN-1 or adult nesidioblastosis GLP-1R imaging is useful whereas in postprandial hypoglycemia in the context of bariatric surgery, GLP-1R imaging is probably not helpful. This review focuses on the potential use of GLP-1R imaging in the differential diagnosis of EHH.

## Introduction

Insulinomas are rare (incidence 1-4/Mio/year), usually benign insulin-secreting neuroendocrine neoplasms (NEN) located in the pancreas (Service* et al.* 1991, Placzkowski* et al.* 2009). They are capable of secreting insulin in an autonomous fashion resulting in life-threatening hypoglycemia. Clinically, these patients present with a panoply of neurological symptoms ranging from simple confusion and neurocognitive deficits to seizures and coma (Valente* et al.* 2018). Importantly, evidence of Whipple's triad (documentation of hypoglycemia in the presence of clinical symptoms with improvement after ingestion of carbohydrates (Whipple & Elliott 1938)) is mandatory in order to pursue to the standardized provocation test, i.e. a 72-h fasting test with monitoring of glucose, insulin and C-peptide levels (Grant 2005).

Insulinomas are the most common cause of the so-called endogenous hyperinsulinemic hypoglycemia (EHH) in adults (Service* et al.* 1991, Grant 2005). The small size of insulinomas (between 1 and 2 cm) challenges the detectability by conventional imaging techniques such as contrast-enhanced CT (ceCT) and contrast-enhanced MRI (Liu* et al.* 2007, Mehrabi* et al.* 2014, Zhu* et al.* 2017). This is due to motion artifacts such as respiratory motion, cardiac pulsation and bowel peristalsis and lack of contrast (Ehman* et al.* 1986). Endoscopic ultrasound is well established in the detection of insulinomas. However, this technique is operator dependent, invasive, and the visualization of the pancreas tail is not always possible (Mehrabi* et al.* 2014, Falconi* et al.* 2016). Since at present surgery remains the only curative treatment, the exact preoperative localization of insulinoma is critical to facilitate pancreas-preserving surgery such as limited segmental resection or enucleation (Falconi* et al.* 2016).

In approximately 25–35% of patients with clinical symptoms and documented EHH, the preoperative localization of insulinomas using conventional imaging procedures such as MRI and ceCT is not possible (Mehrabi* et al.* 2014). In these cases, current recommendations suggest the selective arterial calcium stimulation test with hepatic venous sampling (SACST) (Falconi* et al.* 2016). This test involves selective injection of calcium gluconate into the gastroduodenal, splenic, and superior mesenteric arteries with subsequent sampling of the hepatic venous effluent for insulin (Doppman* et al.* 1991). The pathophysiological background of this test is based on the observation that calcium stimulates the release of insulin from hyperfunctional beta cells (i.e. insulinomas) but not from normal beta cells (Doppman* et al.* 1991). The results of this test enables the surgeon to limit the intraoperative search of the insulinoma to the corresponding arterial territory, which facilitates intraoperative localization. The Mayo clinic series of >200 patients with insulinomas, not localized using conventional imaging procedures, report a sensitivity of >90% using SACST (Placzkowski* et al.* 2009). However, this method determines only the arterial territory and not the tumor itself. In addition, SACST is an invasive procedure with the associated risk for complications, which can result in significant morbidity and even mortality. It is, therefore, only performed in high-volume NEN centers (Placzkowski* et al.* 2009) and there is an unmet need for a non-invasive and reliable method, which is able to selectively and directly localize insulinomas.

## Molecular imaging

Molecular imaging is a relatively new field that emerged from translational research at the intersection of molecular biology and *in vivo* biomedical imaging. It has many applications, mainly in oncology, as many tumors express or activate tumor-specific target molecules or metabolic pathways (Antwi* et al.* 2019*a*). Importantly, the results of basic research define the specific molecular target of a particular tumor (i.e., a receptor, metabolite). The competence of the radiopharmacy is to design, develop and produce the molecule that includes the radioisotope. Finally, the nuclear medicine physician administers the radioisotope-labeled molecule and evaluate the images. Thus, a successful development of a new tracer involves multiple competences and needs an interdisciplinary approach (Antwi* et al.* 2019*a*).

Neuroendocrine neoplasms (NENs) are heterogeneous with respect to the site of origin and metastatic behavior. Insulinomas are part of the about 25% of secreting NEN, the remaining ca. 75% are so-called non-secreting NEN (Dasari* et al.* 2018). NEN in general exhibits an exclusive biologic feature: They homogenously overexpress specific peptide hormone receptors at the surface of the tumors (Reubi & Waser 2003). They are targeted by small regulatory peptides, which have different functions within the gastrointestinal tract, but also within the endocrine system (i.e. pancreatic islets) (Körner 2016). In addition, there are effects of these peptides on the peripheral and CNS (Körner 2016). In tumors, they control mainly hormone secretion and cell proliferation and represent important molecular targets for clinical applications (Reubi & Waser 2003).

Reubi * et al.* have shown that the somatostatin receptors (SSTRs) especially somatostatin subtype 2 receptors (SSTR2) are expressed in high incidence and at high levels in gastroenteropancreatic NEN (Reubi 2003). Consequently, somatostatin receptor scintigraphy using the somatostatin analog octreotide (OctreoScan®) has been established as a diagnostic-staging tool, which is nowadays more and more replaced by ^68^Ga-DOTATOC/-DOTATATE/-DOTANOC PET/CT due to the shorter investigational procedure, the lower radiation burden and the higher sensitivity of the PET modality (Gabriel* et al.* 2007, Wild* et al.* 2013, Etchebehere* et al.* 2014, Sadowski* et al.* 2016). Unfortunately, insulinomas exhibit comparatively low expression of SSTR in contrast to the other secreting and non-secreting NEN (Reubi & Waser 2003). Accordingly,* in vivo* SSTR imaging for insulinomas is often negative (Plockinger* et al.* 2004). Hence, a different molecular target for an imaging modality has been searched for.

An alternative molecule is dihydroxyphenylalanin (DOPA). NENs take up DOPA and the labeled metabolite (^18^F-DOPA) via a specific cell membrane bound transporter for PET imaging. Within the cell, it is decarboxylated to ^18^F-dopamine. Compared with somatotstatin receptor scintigraphy, ^18^F-DOPA PET/CT has a superior performance in imaging NENs (Koopmans* et al.* 2008). However, compared with somatostatin receptor PET/CT, ^18^F-DOPA PET/CT has an inferior performance in patients with NENs (Ambrosini* et al.* 2008). Nevertheless, ^18^F-DOPA PET showed a relatively high sensitivity in congenital hyperinsulinism and adult nesidioblastosis (Stanley 2016).

## Glucagon-like peptide-1 (GLP-1) and glucagon-like peptide-1 receptor (GLP-1 R)

A promising candidate is the incretin glucagon-like peptide-1 (GLP-1) and its respective receptor, the glucagon-like peptide 1 receptor (GLP-1R). This receptor has been cloned approximately 25 years ago (Thorens* et al.* 1993). Similar to the SSTR, it is a member of the class 2 G-protein-coupled receptor family** [Bibr bib51]. Only a single GLP-1R is known so far, which is structurally identical in all tissues (Thorens* et al.* 1993).

The GLP-1R is of clinical interest not only due to its physiologic expression and functions in pancreatic islet cells and its established role in the therapy of type 2 diabetes using GLP-1 analogs (Nauck 2016), but also because of its possible role in cancer. Indeed, the GLP-1R is also overexpressed in insulin-producing islet cell tumors, that is, insulinomas (Reubi & Waser 2003). Importantly, an extensive evaluation concerning the potential of the GLP-1R for targeted tumor imaging confirmed a nearly 100% incidence of GLP-1R expression on benign insulinomas with an about 5x higher density of GLP-1R on insulinomas compared to normal beta cells (Reubi & Waser 2003). The high incidence and density of GLP-1R is an important prerequisite for a successful peptide receptor targeting for diagnostic purposes.

## Radiolabeled GLP-1 analogs

**Figure 1:**
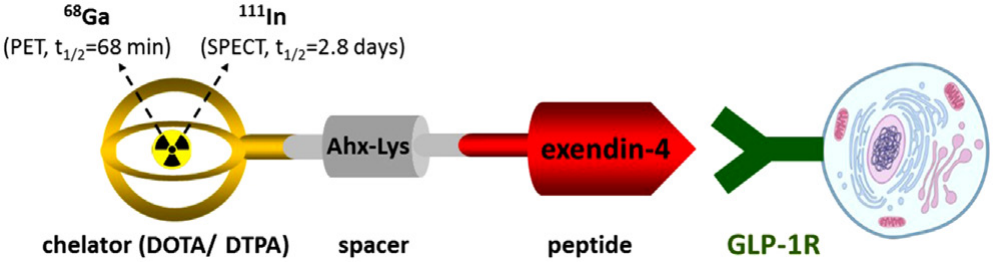
Structure of a peptide-receptor radionuclide targeting glucagon-like peptide-1 receptor for diagnostic administration. The radiopeptide is composed of the specific ligand (exendin-4) bearing a Lysin (Lys) and a spacer (Ahx) linking the ligand to a chelator (DOTA or DTPA) able to be labeled with the radioisotope for SPECT (i.e. ^111^In: gamma-emitter) or PET (i.e. ^68^Ga: positron emitter).

In general, radioactively labeled peptide analogs are pharmaceuticals with favorable biological characteristics. Due to their small size, they show fast diffusion and rapid blood clearance and lack immunogenicity. Moreover, radiopeptides exhibit only rarely severe adverse events (Reubi & Waser 2003). In addition, radiolabeling is feasible, preferably after attaching a chelator to the peptide (Reubi & Maecke 2008) (Fig. 1). Nevertheless, since peptides are physiologically degraded within minutes in the human blood by potent peptidases such as dipeptidyl-peptidase-4 (DPP-4) (Baggio & Drucker 2007), stable peptide analogs have to be used instead in clinical applications. As for GLP-1, a naturally occurring stable analog exists, namely exendin-4, which is a component of the Gila monster venom. It shares 53% homology with GLP-1 and similarly binds to GLP-1Rs, but is resistant to DPP-4 cleavage (Nauck 2009). Exendin-4 is, therefore, a suitable candidate for the development of radiolabeled GLP-1R ligands.

The first radiopeptides tested for *in vivo* GLP-1R targeting were ^125^I-labeled GLP-1 and the GLP-1 analog exendin-3 (Gotthardt* et al.* 2002). However, the low peptide stability of GLP-1 and the low efficiency of radio-iodination of exendin-3 limited their clinical use. Further testing resulted in the development of ^111^In-labeled exendin-4 (Wild* et al.* 2006). Exendin-4 was coupled via the side chain of the Lys to a chelator (DOTA = 1,4,7,10-tetraazacyclododecane-1,4,7,10-tetraacetic acid or DTPA = diethylenetriaminepentaacetic acid) using a spacer (Ahx = aminohexanoic acid) and then labeled with ^111^In (Wild* et al.* 2006) (Fig. 1). These radiopeptides were comprehensively tested *in vitro* and *in vivo* in insulinoma animal models and applied to insulinoma patients (see below). Lately, several studies have been published that describe GLP-1R ligands suitable for PET/CT imaging, such as ^68^Ga-, ^64^Cu- or ^18^F-labeled exendin-4, or for SPECT/CT imaging like ^99m^Tc-labeled exendin-4 (Brom* et al.* 2010, Wu* et al.* 2011, Kiesewetter* et al.* 2012).

## GLP-1R imaging for diagnosis of insulinomas in humans

In 2008 the first two patients with proven EHH underwent GLP-1R scintigraphy. Both patients suffered from severe EHH, and previous conventional imaging or selective arterial stimulation and venous sampling were negative or inconclusive (Wild* et al.* 2008). Importantly, in one of these two patients, SACST indicated the insulinoma in the arterial territory of the A. mesenterica superior. ^111^In-exendin-4 SPECT/CT confirmed the insulinoma in this arterial territory. However, focal ^111^In-exendin-4 uptake was found in the mesenterium below the uncinate process, demonstrating an ectopic localization of an insulinoma (Wild* et al.* 2008). Without the direct localization of the insulinoma using molecular imaging, the therapeutic strategy – based on the result of the SACST – would have been a Whipple’s procedure without removing the ectopic insulinoma. In both patients the GLP-1R positive lesion was surgically resected, and histologic analysis confirmed the insulinoma (Wild* et al.* 2008).

In a proof-of-principle study, ^111^In-DOTA-exendin-4 was administered to a total of six patients all of which presented with neuroglycopenic symptoms due to documented EHH. MRI was capable of localizing a suspicious lesion in only one patient (Christ* et al.* 2009). In contrast, ^111^In-DOTA-exendin-4 scintigraphy correctly detected a histologically proven insulinoma in all six consecutive patients (Christ* et al.* 2009). *In vitro* autoradiography of GLP-1R exhibited a density of GLP-1R in the range as previously described (Reubi & Waser 2003), whereas SSTR expression revealed low density of somatostatin type 1 and 3 receptors in two patients only (Christ* et al.* 2009). Fortunately, background uptake over the whole body is low with the exceptions of the kidneys, which are strongly labeled due to renal excretion of the radioligand. In two patients demarcation between tumors (maximal diameter of 9–11 mm) and kidneys was only possible after late scans indicating an improved tumor to kidney ratio with time, in keeping with the fact that the effective half-life of ^111^In-DOTA-exendin-4 was longer in the tumor (38 – 64 h) than in the kidneys (31.2 – 31.9 h) (Christ* et al.* 2009).

The first prospective multicenter study included 30 patients with proven EHH, which were submitted to ^111^In-DTPA- exendin-4 imaging. Whole-body planar images and SPECT/CT of the abdomen were performed at 4, 24 and in some patients between 72 and 96 up to 168 h post injection (Christ* et al.* 2013). The most important time point was the scan 24 h after injection. In 25 patients, which underwent surgery (with histological analysis as gold standard), ^111^In-DTPA-exendin-4 SPECT/CT correctly detected 19 insulinomas and four additional positive lesions (two islet-cell hyperplasias and two uncharacterized lesions) resulting in a positive predictive value of 83% (Christ* et al.* 2013). ^111^In-DTPA-exendin-4 SPECT/CT had a higher sensitivity (95%) than CT/MRI (47%). Seven patients were operated on because GLP-1R imaging was the only method that showed a suspicious lesion in the pancreas. Finally, five of these patients had a confirmed insulinoma with normalization of hyperinsulinism after surgery, supporting the clinical value of GLP-1R imaging. Also technetium-99m labeled exendin-4 (^99m^Tc-HYNIC-exendin-4, where HYNIC = hydrazinonicotinic acid) scintigraphy and SPECT/CT performed better than established imaging such as CT and somatostatin receptor subtype 2 (SSTR2) imaging in 11 patients with proven EHH (Sowa-Staszczak* et al.* 2013). This is in contrast to findings of Prasad *et al*. (Prasad* et al.* 2016) who reported a sensitivity of >90% for somatostatin receptor PET imaging, possibly due to the higher spatial resolution of PET compared to SPECT.

**Figure 2:**
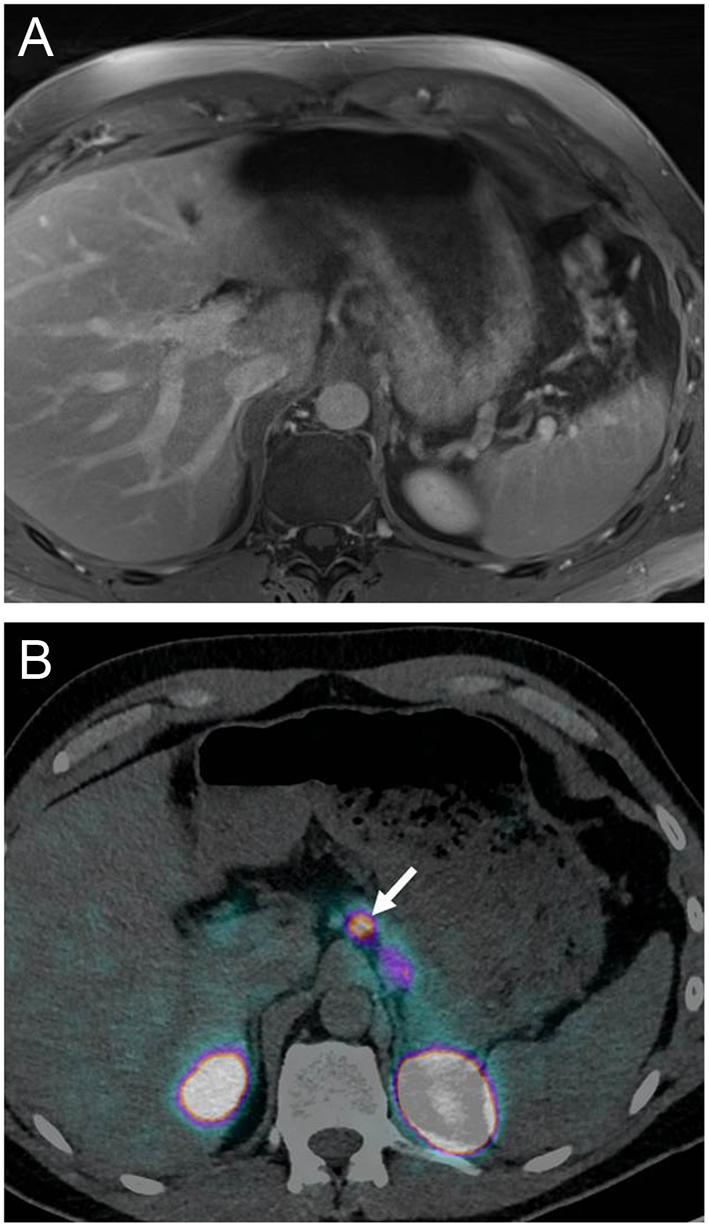
Standardized magnetic resonance imaging and glucagon-like peptide-1 receptor imaging (pancreatic body). Patient with endogenous hyperinsulinemic hypoglycemia without evidence of an insulinoma in prior conventional imaging. (A) Postcontrast T1-weighted images 3 Tesla MRI. (B) PET/CT 2 h after administration of ^68^Ga-DOTA-Exendin-4. On MRI scan no focal lesion was detected, whereas ^68^Ga-DOTA-Exendin-4 PET/CT shows a focal uptake in the body of the pancreas. Histological evaluation confirmed a benign insulinoma in the pancreatic tail.

**Figure 3:**
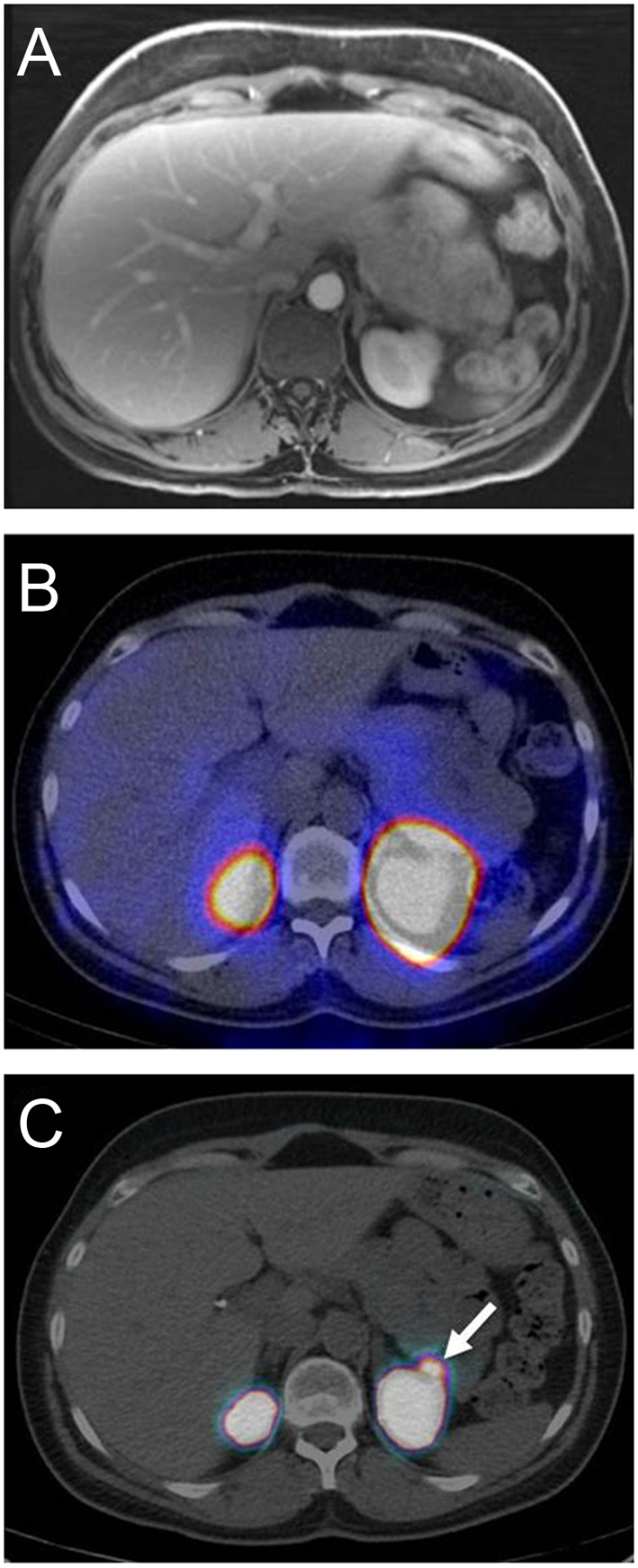
Standardized magnetic resonance imaging and glucagon-like peptide-1 receptor imaging (pancreatic tail). Patient with endogenous hyperinsulinemic hypoglycemia without evidence of an insulinoma in the prior conventional imaging. (A) Crossectional imaging using a 3 Tesla MRI (Siemens). (B) SPECT/CT 72 h after administration of ^111^In-DOTA-exendin-4. (C) PET/CT 2h after administration of ^68^Ga-DOTA-exendin-4. On MRI scan and on ^111^In-DOTA-exendin-4 no focal lesion was detected whereas ^68^Ga-DOTA-exendin-4 PET/CT shows a focal uptake in the tail of the pancreas (white arrow). Histological evaluation confirmed a benign insulinoma in the pancreatic tail.

Since PET has several advantages over SPECT (i.e. higher spatial resolution, shorter investigational time, lower radiation burden), ^68^Ga-DOTA-exendin-4 PET and ^111^In-exendin-4 SPECT were tested in a proof-of-principle study in five patients with EHH and negative or controversial findings on conventional imaging (Antwi* et al.* 2015). The results of this proof-of-principle study showed a better performance of ^68^Ga-DOTA-exendin-4 PET/CT compared with ^111^In-DOTA-exendin-4 SPECT/CT (Antwi* et al.* 2015). Furthermore, Luo *et al*. found an excellent sensitivity of ^68^Ga-NOTA-exendin-4 PET/CT (NOTA = 1,4,7-triazacyclononane-1,4,7-triacetic acid) of more than 97% in 45 patients with EHH (Luo* et al.* 2016):

Based on these promising data, a prospective clinical study including 52 consecutive patients with a positive Whipple’s trias and a proven EHH was conducted at the University Hospital Basel (Antwi* et al.* 2018). The aim was to compare the diagnostic accuracy and clinical impact of ^68^Ga-DOTA-exendin-4 PET/CT in comparison with ^111^In-DOTA-exendin-4 SPECT/CT. In addition, this time a standardized conventional imaging procedure (3-Tesla MRI) was included in the study protocol since our previous experiences with conventional imaging procedure performed at the respective referring hospital showed potential lesions in less than 50% of the patients, which is clearly lower than reported in the literature (Mehrabi* et al.* 2014). In this prospective study the accuracy of ^68^Ga-DOTA-exendin-4 PET/CT was 93.9% which was significantly higher than for SPECT/CT (67.5%) and MRI (67.6%) (Antwi* et al.* 2018) (Fig. 2 and Table 1). In addition, reader agreement between three independent radiology and nuclear medicine readers was higher for PET/CT (89.5%), than for ^111^In-DOTA-exendin-4 SPECT/CT (75.7%) and MRI (71.1%) (Antwi* et al.* 2018). This may be attributed to the higher tumor-to-background ratio of PET/CT in comparison to SPECT/CT or low signal to noise ratio in MRI due to motion artifacts (Antwi* et al.* 2018). The lower accuracy of SPECT/CT in comparison to PET/CT was mainly due to insulinomas located in the pancreatic tail in close proximity of the left kidney as SPECT/CT was not able to distinguish the insulinoma from the kidney uptake (Fig. 3). This confirmed that higher spatial resolution, higher scanner sensitivity, and higher tumor-to-background ratio aids visual assessment of PET/CT. Importantly the kidney uptake of radiolabeled exendin-4 can be reduced by prior infusion of succinylated gelatin (colloidal volume expander) (Buitinga* et al.* 2019) which may help uncover the insulinoma in the pancreatic tail also by PET/CT (Antwi* et al.* 2019*b*). Taking the lower radiation burden and the shorter imaging procedure into account makes ^68^Ga-DOTA-exendin-4 PET/CT the preferred procedure for the localization of insulinomas.

**Table 1:** Comparison of GLP-1R imaging, standardized contrast enhanced (ce) MRI and prior non-standardized ceCT/MRI.

	^68^Ga-exendin-4 PET/CT	Standardized ceMRI (3 Tesla)	^111^In-exendin-4 SPECT/CT	Prior not standardized ceCT/MRI
Sensitivity (pooled analysis)	94%	68%	68%	41%
Sensitivity range (3 readers)	90–100%	67–90%	67–81%	N/A
Positive predictive value	99%	97%	96%	100%

Comparison of GLP-1R imaging, standardized contrast enhanced (ce) MRI and non-standardized ceCT/ceMRI in patients with suspected insulinoma and available reference standard (surgery and normalization of blood glucose levels). Imaging performance is given as the averages (95% CI) of three readings by three independent readers except baseline ceMRI and ceCT which were performed and interpreted at referring centres. SPECT/CT results are based on 24 h and 72 h reading.

N/A, not applicable. Some data from Antwi *et al*. (2018).

## Non-GLP-1R PET/CT imaging for diagnosis of insulinomas in humans

Somatostatin receptor (SSTR) PET/CT with ^68^Ga-DOTATATE, ^68^Ga-DOTATOC and ^68^Ga-DOTANOC as well as ^18^F-fluorodopa (^18^F-DOPA) PET/CT are described to be effective as well in the localization of insulinomas. For example, ^68^Ga-DOTATATE or ^68^Ga-DOTATOC PET/CT detected insulinomas or nesidioblastosis in 11/13 patients (detection rate of 85%) in a retrospective study (Prasad* et al.* 2016). In the same study CT was nearly as good as SSTR PET/CT, with a detection rate of 77% (10/13 patients), indicating that these were not particularly difficult cases. On the other hand GLP-1R PET/CT with ^68^Ga-NODAGA-exendin-4 detected insulinomas with a much higher sensitivity than SSTR PET/CT (93.5 vs 61.3%) in a prospective comparison in 31 patients with biochemically proven hyperinsulinemic hypoglycemia (Boss *et al.* 2019). These results are in line with* in vitro* autoradiography studies in 26 insulinoma tissue samples: GLP-1R was expressed in 24/26 samples (92%) at a high density, whereas SSTR2 was expressed in 18/26 samples (69%) at a moderate to high density (Reubi & Waser 2003).

Kauhanen *et al*. detected insulinomas or nesidioblastosis in 9/10 patients (90%) with ^18^F-DOPA PET (Kauhanen* et al.* 2007). These initially excellent results could not be repeated by other groups: Nakuz *et al*. detected the insulinoma in 5/10 patients (50%) with ^18^F-DOPA PET, whereas Imperiale *et al*. showed somewhat better results with carbidopa pretreatment that seems to reduce the physiological uptake of ^18^F-DOPA in the pancreas (insulinoma detection was 73%) (Imperiale* et al.* 2015, Nakuz* et al.* 2018). The evidence level for direct comparison of GLP-1R imaging and ^18^F-DOPA PET is scarce: we showed in a subpopulation of our latest prospective GLP-1R imaging study who received both PET/CT scans (5/52 patients) a better performance of ^68^Ga-DOTA-exendin-4 PET/CT compared to ^18^F-DOPA PET/CT without carbidopa (insulinoma detection rate was 93% versus 0%) (Antwi* et al.* 2018). Table 2 summarizes the current literature about the performance of different modalities in the localization of insulinomas.

**Table 2:** Performance of different modalities in the localization of insulinomas.

	Sensitivity	References^a^
MRT	56–90%	(Morganstein* et al.* 2009, Luo* et al.* 2016, Wei* et al.* 2016, Zhu* et al.* 2017, Antwi* et al.* 2018, Fu* et al.* 2018)
Multiphase CT	44–74%	(Morganstein* et al.* 2009, Tavcar* et al.* 2014, Imperiale* et al.* 2015, Téllez-Ávila* et al.* 2015, Luo* et al.* 2016, Moreno-Moreno* et al.* 2016, Morera* et al.* 2016, Prasad* et al.* 2016, Wei* et al.* 2016, Zhu* et al.* 2017, Antwi* et al.* 2018, Fu* et al.* 2018, Nakuz* et al.* 2018, Andreassen* et al.* 2019, Boss* et al.* 2019)
EUS	70–100%	(Morganstein* et al.* 2009, Tavcar* et al.* 2014, Imperiale* et al.* 2015, Téllez-Ávila* et al.* 2015, Luo* et al.* 2016, Moreno-Moreno* et al.* 2016, Morera* et al.* 2016, Prasad* et al.* 2016, Wei* et al.* 2016, Zhu* et al.* 2017, Antwi* et al.* 2018, Fu* et al.* 2018, Nakuz* et al.* 2018, Andreassen* et al.* 2019, Boss* et al.* 2019)
ASVS	65–100%	(Morganstein* et al.* 2009, Tavcar* et al.* 2014, Imperiale* et al.* 2015, Téllez-Ávila* et al.* 2015, Luo* et al.* 2016, Moreno-Moreno* et al.* 2016, Morera* et al.* 2016, Prasad* et al.* 2016, Wei* et al.* 2016, Zhu* et al.* 2017, Antwi* et al.* 2018, Fu* et al.* 2018, Nakuz* et al.* 2018, Andreassen* et al.* 2019, Boss* et al.* 2019)
SSTR SPECT/CT	20–33%	(Morganstein* et al.* 2009, Tavcar* et al.* 2014, Imperiale* et al.* 2015, Téllez-Ávila* et al.* 2015, Luo* et al.* 2016, Moreno-Moreno* et al.* 2016, Morera* et al.* 2016, Prasad* et al.* 2016, Wei* et al.* 2016, Zhu* et al.* 2017, Antwi* et al.* 2018, Fu* et al.* 2018, Nakuz* et al.* 2018, Andreassen* et al.* 2019, Boss* et al.* 2019)
SSTR PET /CT	61–87%	(Prasad* et al.* 2016, Boss* et al.* 2019)
GLP-1R SPECT/CT	69%	(Antwi* et al.* 2018)
GLP-1R PET/CT	94–98%	(Luo* et al.* 2016, Antwi* et al.* 2018)
^18^F-DOPA PET/CT^b^	50–73%	(Imperiale* et al.* 2015, Nakuz* et al.* 2018)

Table 2 summarizes the performance of different modalities in the localization of insulinomas.

^a^References include all English written literature not older than 6 years with more than 12 patients using the following keywords: respective localization modality and insulinoma or endogenous hyperinsulinemic hypoglycemia. ^b^Studies with less than 12 insulinoma patients were included here since larger ^18^F-FDOPA PET/CT studies are not performed yet.

## Malignant insulinomas

Only about 10% of insulinomas present with malignant behavior at diagnosis, i.e. metastasis at diagnosis (Grant 2005, Placzkowski* et al.* 2009). Clinically, patients with malignant insulinomas present with similar signs and symptoms as patients with other differential diagnosis of EHH, notably benign insulinomas. The biochemical work-up is identical as in benign insulinomas, i.e. positive Whipple’s triads and documentation of EHH (Falconi* et al.* 2016). However, conventional imaging (ceCT or MRI) usually show a pancreatic NEN often with peri-pancreatic suspicious lymph nodes and hepatic metastasis (Placzkowski* et al.* 2009). Complete surgical resection of all tumors is often difficult and prognosis remains relatively poor, with a 5-year survival of 55.6% and 10-year survival of 29% (Service* et al.* 1991, Grama* et al.* 1992, Lepage* et al.* 2010). Accurate assessment of the extent of the disease (’staging’) is essential, primarily because pre-interventional localization of all lesions facilitates surgery or local-ablative approaches (Falconi* et al.* 2016). Remarkably, intervention aiming at reducing the tumor burden (without a curative strategy) is often indicated because of the sometimes severe and life-threatening hypoglycemia (Falconi* et al.* 2016). This is of particular importance since medical therapy for EHH is sometimes difficult and often needs a combined treatment including Diazoxid, Somatostatin analogs, Everolimus and Prednisone in addition to regular ingestion (or even infusion) of glucose with the associated increase in body weight (Falconi* et al.* 2016).

In order to allow for a comprehensive staging in patients with suspected malignant insulinomas, the incidence and density of peptide receptor status in 11 patients with malignant insulinomas were established *in vivo* using GLP-1R and SSTR2 imaging and/or *in vitro* using autoradiography of the tumor samples (Wild* et al.* 2011). The results indicate that GLP-1 receptor targeting was positive in four of 11 (36%), and SSTR2 imaging was positive in eight of 11 patients (64%). In only one patient, both receptors were expressed (Wild* et al.* 2011). Importantly, in all patients, one of these two receptors was overexpressed. Our data indicate that, in contrast to benign insulinomas, malignant insulinomas often lack GLP-1 receptors but express SSTR2. This observation is clinically relevant since in case of a positive SSTR2 imaging a peptide radionuclide receptor therapy (PRRT) using ^177^Lu-DOTATOC or ^177^Lu-DOTATATE (Lutathera®) can be performed (van Schaik* et al.* 2011, Wild* et al.* 2011). Unfortunately, due to a high renal uptake and the potential associated side effects, PRRT using exendin-4 is not an option in malignant insulinoma expressing GLP-1Rs (Wild* et al.* 2011).

It is conceivable that the expression of the corresponding receptors is related to the biological behavior of the tumor (benign vs malignant); however, the exact role of these receptors in the malignant or benign course of the insulinoma is unclear and remains to be established.

## Special cases

Endogenous hyperinsulinemic hypoglycemia in the context of multiple endocrine neoplasia type 1 (MEN-1)

MEN-1 is an autosomal dominant inherited tumor syndrome caused by heterozygous mutations in the *MEN-1* tumor suppressor gene with an incidence of 1:50’000. More than 80% of these patients develop multifocal secreting or non-secreting pancreatic NEN during their lifetime. Pancreatic NEN and their malignant course is one of the major cause of premature death in these patients (Wilkinson* et al.* 1993, Dean* et al.* 2000, Romanet* et al.* 2019). Most frequently, the secreting NEN in MEN-1 include duodenal gastrinomas causing Zollinger Ellison Syndrome and pancreatic insulinomas causing EHH with the corresponding clinical symptoms (Service* et al.* 1991). As in sporadic cases, surgery is the cornerstone of therapy since current medical treatment options do not provide a permanent cure, and thus should be reserved for the perioperative period or for patients who cannot undergo surgery (Vezzosi* et al.* 2008).

Previous studies have proposed aggressive resection of pancreatic NEN identified by conventional imaging (Akerström* et al.* 2002, Hausman* et al.* 2004) which is associated with significant mortality and long-term morbidity including exocrine and endocrine insufficiency (Kahl & Malfertheiner 2004, Falconi* et al.* 2008). Recent reports suggest that non-secreting NEN in MEN-1 patients, which are smaller than 20 mm, rarely develop metastases (Triponez* et al.* 2006, Partelli* et al.* 2016) and an aggressive surgical intervention has not shown any survival benefit for patients with non-secreting pancreatic NEN ≤ 20 mm who had regular follow-up with conventional imaging (Triponez* et al.* 2006, Nell* et al.* 2018). Consequently, in patients with non-metastatic pancreatic NEN the international guidelines suggest resection of symptomatic insulinomas of any size, but resection of non-secreting NEN only above a size ≥20 mm (Vinik* et al.* 2010, Falconi* et al.* 2016).

In clinical routine conventional imaging including ceCT and MRI are reliable tools to assess the size of pancreatic NEN, in particular for lesions >20 mm. In the context of MEN-1 patients, when multiple pancreatic NEN are detected, these investigations remain insufficient due to mainly two reasons. First, the usually small size of insulinomas (<20 mm) (Liu* et al.* 2007, Mehrabi* et al.* 2014) makes them susceptible to motion artifacts, such as respiratory motion, cardiac pulsation and bowel peristalsis, and thus limits their detectability (Ehman* et al.* 1986). Secondly, morphological imaging as well as SSTR2 imaging is not able to differentiate insulin-secreting NEN from non-secreting pancreatic NEN. In view of the often multiple pancreatic NENs visualized on conventional imaging in the context of *MEN-1* patients, the exact localization of the insulin-secreting PanNET is critical for the surgical strategy so as to avoid unnecessary morbidity due to too extensive surgery.

In the context of *MEN-1*, the biological characteristics of insulin-secreting NEN is not established. To improve the knowledge we retrospectively analyzed data of six patients with EHH in the context of MEN-1 who underwent ^68^Ga-DOTA-exendin-4 PET/CT (Antwi* et al.* 2019*c*). The results indicate that ^68^Ga-DOTA-exendin-4 PET/CT is indeed able to localized insulinomas in *MEN-1* patients with high accuracy, i.e. in the context of MEN-1 insulinomas overexpress GLP-1R as in patients with sporadic insulinomas (Antwi* et al.* 2019*c*). It is a useful and reliable imaging technique to selectively identify insulinomas within the numerous pancreatic NEN in patients with MEN-1 (Antwi* et al.* 2019*c*). The careful interpretation of a morphological modality (MRI) including the size of lesions (below or greater than 20 mm) in combination with a functional imaging technique (GLP-1R PET/CT) is able to guide the surgical strategy, thereby avoiding unnecessary pancreatic resections (Antwi* et al.* 2019*c*).

GLP-1R PET in combination with contrast-enhanced CT as a one-stop shot is a recommended combined imaging modality here. Another alternative is the use of an integrated PET/MRI scanner. However, for the latter there are only case reports in the literature indicating that ^68^Ga-DOTA-Exendin PET-MRI may help in precisely localizing the culprit lesion, whereas contrast-enhanced CT and ^18^F-DOPA PET failed to do so (Sood* et al.* 2019). For the moment, data are too scare to draw firm conclusions.

## Endogenous hyperinsulinemic hypoglycemia in the context of adult nesidioblastosis and postprandial hypoglycemia in the context of bariatric surgery

The term ‘nesidioblastosis’ has been used to denote characteristic histopathological findings such as neogenesis from pancreatic ductal epithelium and Beta-cell hyperplasia and hypertrophy (Anlauf* et al.* 2005). This entity was first described in children and neonates and is a differential diagnosis of EHH. The pathophysiological mechanisms in adults are unclear (Anlauf* et al.* 2005). Clinically, the distinction between insulinoma and nesidioblastosis is usually not possible. The preoperative distinction between insulinoma and nesidioblastosis is of primordial importance since the therapeutical strategies are different, ranging from an enucleation in the case of an insulinoma up to a 70–80% pancreatectomy in the case of nesidioblastosis. Furthermore, a focal nesidioblastosis may have a different surgical approach than a nesidioblastosis of the whole pancreas. Preoperative imaging including MRI, CT, and endosonography is not diagnostic and invasive investigation using selective arterial calcium stimulation and venous sampling (ASVS) is recommended with an increase of insulin levels upon calcium stimulation in several or all arterial territory (Thompson* et al.* 2015).

In the context of the prospective study (Antwi* et al.* 2018) one patient with a focal adult nesidioblastosis was non-invasively suspected using ^68^Ga-DOTA-exendin-4 PET/CT and histologically proven following pancreatic surgery (Christ* et al.* 2015). The molecular rationale for the positive ^68^Ga-DOTA-exendin-4 PET was found in the performed autoradiography study that demonstrated a more than 3-time higher density of GLP-1R in the islets of this nesidioblastosis patient when compared with islets of a normal pancreas (Christ* et al.* 2015). The immunohistochemical findings suggest that these GLP-1Rs were mainly expressed in the insulin-producing islet cells (Christ* et al.* 2015). The increased number of GLP-1R in Beta-cells is further potentiated by a considerable increase in number and size of the islets in this nesidioblastosis case. Importantly, the density of GLP-1R in the nesidioblastosis of the pancreas was higher than in normal pancreatic islets, but lower than in benign insulinomas, reflecting the moderate intensity of the *in vivo* scan in this patient and corroborating the excellent correlation between* in vitro* and* in vivo* detection of GLP-1Rs (Christ* et al.* 2009, 2015). Since adult nesidioblastosis can be a focal disease, a non-invasive preoperative tool like ^68^Ga-DOTA-exendin-4 PET may be helpful in determining the surgical strategy, but clearly more evidence is needed in this field, in particular it would be of interest to investigate children with genetically determined nesidioblastosis. Similarly, the pathophysiological role of GLP-1 and GLP-1Rs in the context of nesidioblastosis has to be elucidated.

A distinct clinical syndrome is that of postprandial hypoglycemia, typically occurring after gastric bypass surgery for obesity (Reubi* et al.* 2010). While the specific pathophysiology of this syndrome remains unclear, carbohydrate intake results in rapid elevations of plasma glucose, insulin, and C-peptide levels, with subsequent development of hypoglycemia during the later postprandial period (Patti* et al.* 2005, Service* et al.* 2005, Meier* et al.* 2006, Goldfine* et al.* 2007). In some individuals with this syndrome, diffuse islet cell hyperplasia and expansion of beta-cell mass has been observed (Patti* et al.* 2005, Service* et al.* 2005), in other studies, increased nuclear size relative to body mass, but not hyperplasia has been reported (Meier* et al.* 2006). However, in a small subset of individuals, this form of hypoglycemia is so severe that nutritional as well as pharmacological interventions are without any effect and partial pancreatectomy has to be performed for symptom control (Patti* et al.* 2005, Service* et al.* 2005). In fresh frozen pancreatic tissue samples from six gastric-bypass surgery patients suffering from severe postprandial hyperinsulinemic hypoglycemia GLP-1R density was evaluated *in vitro* using the standardized autoradiography method and compared with normal pancreas and with pancreatic insulinoma tissues (Reubi *et al.* 2010). This analysis revealed a mean density value of GLP-1R,s, which was not statistically different from normal pancreatic beta-cells (Reubi* et al.* 2010). Therefore, postprandial EHH in the context of post-bariatric surgery is not accompanied by overexpression of GLP-1R in individual islets and these patients are, therefore, probably not candidates for GLP-1R imaging *in vivo* using radiolabeled exendin-4. However, these findings have to be confirmed *in vivo.*

## Side effects and limitations

There were no severe adverse events by administrating indium-111, technetium-99m and gallium-68 labeled exendin-4. Mild nausea was frequently reported, in particular with DOTA as a chelator for the radioisotope, less with DTPA. The effects seem to be more pronounced with ^111^In-DOTA-exendin-4 than with ^68^Ga-DOTA-exendin-4 (Christ* et al.* 2013, Antwi* et al.* 2018).

Interestingly, after the injections of the tracer molecule, blood glucose levels often decreased and had to be monitored. Usually, glucose infusion had to be administered because of the significant decrease in glucose concentrations following tracer administration (Christ* et al.* 2013, Antwi* et al.* 2018). The pathophysiological background for this finding is ill-defined since exendin-4 administration in type 2 diabetic patients does usually not lead to hypoglycemic events (Nauck 2016).

 Overall we included nearly 90 patients with potential insulinomas in prospective trials. The inclusion criteria were always identical: positive Whipple’s trias, neuroglycopenic symptoms and a biochemically documented EHH (usually a fasting test with inappropriate insulin and C-peptide levels) (Christ* et al.* 2009, 2013, Antwi* et al.* 2015, 2018). In this context, it is important to mention that a biochemically proven EHH is very specific for insulinomas (Cryer* et al.* 2009) with only few false-positive results. We did not assess patients without biochemically proven EHH who may have false-positive results. It was therefore not possible to evaluate the specificity of GLP-1R imaging.

Reubi & Waser (2003) assessed GLP-1R in different tumor and non-neoplastic tissue. They report (Reubi & Waser 2003) – amongst others – the presence of GLP-1R on the Brunner’s gland in the duodenum. Since these glands are in close proximity to the head of the pancreas, careful interpretation of the GLP-1R imaging has to be performed. The co-registration of a low dose CT with GLP-1R SPECT of PET images usually helps to differentiate between an insulinoma and Brunner’s glands.

## Future developments

Over the last 15 years a number of radiolabeled exendin conjugates have been developed and evaluated preclinically and a number of them clinically (Jansen* et al.* 2019). With focus on further improving preoperative localization, but also therapeutic interventions, effort is still put in both directions. The major drawback of radiolabeled exendin-4 is the high kidney accumulation, which might limit in some cases, accurate diagnosis – for example in the tail region of the pancreas – and in addition the application of targeted radionuclide therapy. A number of strategies were tested to circumvent renal accumulation (Jansen* et al.* 2019), with the most recent ones being: (a) the use the nephroprotective agent succinylated gelatin (Gelofusine©), which combined with ^111^In-exendin-4 in humans reduced kidney uptake (Buitinga* et al.* 2019), (b) conjugation of an albumin-binding moiety (ABM) to the radiolabeled exendin-4, which resulted in a significant reduction of kidney uptake while retaining its high affinity and specificity to GLP-1R in a preclinical model (Kaeppeli* et al.* 2019) and (c) introduction of a cleavable linker between the peptide sequence and the chelator, which is cleaved by brush border membrane enzymes allowing this way rapid excretion from urine, thus significantly reducing renal uptake, in a preclinical model (Zhang* et al.* 2019).

Focusing more on therapy, an interesting development is the bimodal imaging probes (PET/fluorescence) in an attempt to improve intraoperative delineation of the tumor, and complete resection of the insulinoma, via fluorescence-guided surgery, while preserving healthy pancreatic tissue. The conjugation of a near-infrared fluorescent dye and a chelator for labeling with ^64^Cu to exendin-4 provided such a bimodal imaging probe able to visualize small xenografts (<2 mm) with PET and individual pancreatic islets with fluorescence in preclinical settings (Brand* et al.* 2014). In addition, targeted photodynamic therapy (tPDT) via exendin-4 has been proposed as an alternative therapeutic intervention. In this context, a conjugate of exendin-4 with a photosensitizer caused efficient and specific cell death of GLP-1R expressing cells *in vitro* and *in vivo* on mice islets (Boss & Brom 2018).

## Conclusion

Receptors for the incretin glucagon-like peptide-1 (GLP-1R) are overexpressed in selected types of human tumors and may, therefore, play an increasingly important role in gastrointestinal NEN management. Targeting GLP-1R with GLP-1R SPECT/CT and PET/CT offers a new approach that permits the successful localization of small benign insulinomas preoperatively. Since virtually all benign insulinomas express GLP-1Rs and the clinical experience is very encouraging, it is likely that this approach – if widely available – will affect the algorithm of preoperative localization of suspected insulinoma, thereby avoiding the determination of the arterial territory of the insulinoma using the invasive SACST. We, indeed, believe that GLP1-R imaging will lead to the end of the selective calcium stimulation test with venous sampling.

In contrast to benign insulinomas, where the exact localization of the tumor is the primary goal, the clinical challenge in metastasizing insulinomas is to define the extension of disease and – if possible – offer a targeted therapy (peptide receptor radionuclide therapy; PRRT). Contrary to benign insulinomas malignant insulinomas more often express SSTR2 than GLP-1R. Importantly, one of the two receptors seems to be always expressed. The respective role of these receptors with regard to biological behavior is not established.

Data in patients with EHH in the context of MEN-1 indicate that GLP-1R PET/CT is a very sensitive method to preoperatively localize insulin-secreting NEN. This is of particular importance since patients with MEN-1 usually present with multiple pancreatic lesions, and current recommendations suggest to resect only lesions >20 mm or hormone-secreting NEN. GLP-1R PET/CT allows to selectively localize the insulin-secreting NEN, which is usually <20 mm.

In pancreatic nesidioblastosis, GLP-1R expression lies in the range of normal beta-cell and benign insulinomas. Preliminary data indicate that GLP-1R PET/CT may be an appropriate tool to diagnose this condition. In contrast, current evidence suggests that in patients suffering postprandial EHH following bariatric surgery, GLP-1R imaging is not a suitable diagnostic tool.

## Declaration of interest

The authors declare that there is no conflict of interest that could be perceived as prejudicing the impartiality of this review.

## Funding

The study was supported by the Swiss National Science Foundation (grant number 320030-152938) and the Desirée and Niels Yde’s Foundation (grant number 389-12) which had no role in study design, data collection, analysis, interpretation, or writing of the report.
